# Immunization with a fusion protein vaccine candidate generated from truncated peptides of human enterovirus 71 protects mice from lethal enterovirus 71 infections

**DOI:** 10.1186/s12985-020-01328-8

**Published:** 2020-04-22

**Authors:** Jiangning Liu, Binbin Zhao, Ling Xue, Jing Wu, Yanfeng Xu, Yongdong Liu, Chuan Qin

**Affiliations:** 1 NHC Key Laboratory of Human Disease Comparative Medicine, Beijing Key Laboratory for Animal Models of Emerging and Reemerging Infectious Diseases, Beijing Engineering Research Center for Experimental Animal Models of Human Critical Diseases, Institute of Laboratory Animal Science, CAMS&PUMC, Beijing, 100021 People’s Republic of China; 2 National Key Laboratory of Biochemical Engineering, Institute of Process Engineering, Chinese Academy of Sciences, Beijing, 100190 People’s Republic of China; 3 University of Chinese Academy of Sciences, Beijing, 100049 People’s Republic of China

**Keywords:** Human Enterovirus 71, Vaccine candidate, Fusion protein, Mouse model, Infection

## Abstract

**Background:**

Prophylactic vaccines are critical in preventing hand, foot, and mouth disease (HFMD) primarily caused by human enterovirus 71 (EV71) infection. Children aged less than 5 years are especially susceptible to EV71 infections. In addition to the development of vaccines containing the inactivated virus, those containing virus-like particles (VLPs) with repeated antigens also constitute an effective preventive strategy for EV71 infections, with safety and productivity advantages. We previously developed a fusion protein composed with truncated peptides of the EV71 capsid protein, which assembled into spherical particles. This study aimed to assess the immunoprotective effects of this fusion protein as a vaccine candidate in a mouse model of EV71 infection.

**Methods:**

To evaluate the protective effect of fusion protein vaccine candidate, neonatal mice born by immunized female mice, as well as normal neonatal mice immunized twice were infected with EV71 virus. Whereafter, the survival rates, clinical scores and viral loads were measured.

**Results:**

The high dosage and booster immunization helped induce specific serum antibodies with high neutralization titers, which were transferred to neonatal mice, thereby facilitating effective resistance towards EV71 infection. An active immune response was also observed in neonatal mice which generated following immunization.

**Conclusions:**

The present results suggest that this fusion protein is a suitable vaccine candidate in treating EV71 infections.

## Background

Hand, foot, and mouth disease (HFMD) is extremely contagious and prevalent among infants and children aged less than 5 years. Human enterovirus 71 (EV71) has been considered the major pathogen for HFMD, having triggered several disease outbreaks worldwide with high morbidity and mortality rates since its initial isolation in 1969 [1–5]. Multiple countries in America, Europe, Australia and Asia had seen the EV71 epidemic since 1970s [2–5]. During 1997 and 1998, several EV71 outbreaks occurred in Asia, including Sarawak, Malaysia, Japan and Taiwan, while fatal cases were reported [6–9]. The latest large Asian epidemic was in mainland China during 2007 and 2008, with 488,955 cases as well as 126 deaths reported [10, 11]. Since the establishment of national surveillance system in 2008, more than 15 million HFMD cases were reported according to the China National Center for Disease Control (CDC) by 2015, with over 3400 deaths. EV71 predominated among the laboratory-confirmed cases, especially for the severe and fatal cases [12, 13]. Meanwhile, various EV71 vaccines have been developed , and since 2015 three EV71 vaccines have been approved by Chinese FDA to prevent the continuous epidemics [14–16], which were separately developed by Institure of Medical Biology, Chinese Academy of Medical Sciences (CAMS, Kunming Institute), Sinovac Biotech Co., Ltd., and China National Biotech Group. All approved vaccines comprise inactivated viruses with the obvious advantage of almost complete immune protection; in fact, the vaccines are proved to protect over 90% vaccinated children from EV71-related HFMD or herpangina [10, 14, 15, 17].

The virus-like particles (VLPs), containing repeated antigens but excluding the infective genetic material, are developed as potential vaccine candidates due to their safety and efficacy according to animal experiments [18–22]. There’re also two inspiring examples, VLP-based hepatitis B virus and human papillomavirus vaccines, which have been successfully commercialized [23, 24]. Recombinant EV71 VLPs can resemble the natural virus structure in terms of their capsid proteins. Immunization with such VLPs can induce EV71-specific immune response with high neutralizing antibody titers and increased cytokines in mouse model [18, 25–27]. Considering the EV71 inactivated whole-virus vaccines are unable to prevent infection caused by other major agents of HFMD, such as CA16, CA10 and CA6, multivalent vaccines deriving from VLPs are regarded as another option for disease prevention [12, 24].

We previously found that a vaccine comprising a combination of four peptides from EV71 capsid protein (VP1–VP3) (Vac6, including P_70–159_, P_140–249_, P_324–443_, and P_746–876_ peptides) effectively elicited the production of neutralization antibodies in female mice and adequately protected neonatal mice against EV71 infection [28]. Furthermore, we engineered these peptides into one fusion protein (including P_70–249_, P_324–443_ and P_746–876_), using a prokaryotic expression system. The expressed fusion protein finally assembled into sphere-like particulates (VLPs) of 25–40 nm, and were proved to induce humoral and cell-mediated immunity effectively in mice after vaccination, with the detection of high-titer antibodies and promoted splenocyte proliferation and cytokine secretion [18]. In this study, we further investigated the immunoprotective effects of this fusion protein, using a mouse model of EV71 infection, including its effects on the production of neutralizing antibodies, the immunoprotective role of maternal antibodies in neonatal mice, and the protection of active immunization in neonates.

## Methods

### Virus and cell culture

Human rhabdomyosarcoma cells (RD) were cultured in Dulbecco’s modified Eagle’s medium (DMEM) supplemented with 10% fetal bovine serum (FBS) [28]. Clinically isolated EV71 strains FY0805 (GenBank accession no. HQ882182) and a mouse-adapted EV71 strain MP10 (GenBank accession no. HQ712020) derived from FY0805 [29] were cultured along with RD cells, respectively. The 50% tissue culture infectious dose (TCID_50_) was determined by observing CPE using Reed-Muench method [30]. Furthermore, working stocks of EV71 containing 10^8^ TCID_50_/mL were prepared for subsequent experiments.

### Preparation of vaccine candidates containing the fusion protein

The design, expression, purification, and assembly of EV71 fusion protein as a vaccine candidate was performed as described previously [18]. In brief, a DNA fragment encoding three truncates of EV71 capsid protein (180 amino acids located at N-terminal of VP2, 120 amino acids located at N-terminal of VP3, and 131 amino acids located at C-terminal of VP1) were amplified via fusion PCR and ligated at the *Nde*I and *Eco*RI sites of pET30a(+) under the control of the T7 promoter, and two flexible peptides of (Gly_4_Ser)_3_ were adopted to ligate the three fragments together to form a 48-kDa fusion protein. The targeted protein was expressed in *Escherichia coli* BL21 (DE3), upon induction with 1 mM isopropyl-D-thiogalactopyranoside, as inclusion bodies, which were solubilized and denatured in the denaturant buffer containing guanidine chloride. Thereafter, it was purified via a series of ions exchange in urea buffer, while the additive in solution was eliminated using a desalting column. Thereafter, 1 mM CaCl_2_ was added into the desalted sample to initiate the assembly of VLPs. Finally, the fusion protein was stored in 10 mM Glycine-NaOH buffer (pH = 8.0) with 5% glycerol [18].

### Immunization of mice

BALB/c mice were obtained from Beijing HFK Bioscience Co., LTD. The mice were bred in an AAALAC-accredited facility, while protocols were approved by the Animal Care and Use Committee of the Institute of Laboratory Animal Science of Chinese Academy of Medical Sciences (ILAS-PG-2015-014).

For immunization, a commercial Alu-Vac 15 adjuvant (Serva, Germany), which contains 15 mg/mL of aluminum hydroxide was formulated with the purified and assembled fusion protein. Briefly, the fusion protein was diluted to 200 μg/mL, 40 μg/mL, and 10 μg/mL in a volume of 50 μL (which corresponds to 10 μg, 2 μg, and 0.4 μg antigen for each mouse), which was mixed with 10-fold diluted Alu-Vac 15 adjuvant at a volumetric ratio of 1: 1 in accordance with the manufacturer’s instructions. Heat-inactivated EV71 (FY0805) was dissolved at 5.0 × 10^7^ TCID_50_/mL in the same solution and formulated as the positive antigen [18]. Lysates from *E. coli* without fusion protein genes were used as negative controls. Six mice were immunized for each group. The strong cross-reactivity inducing peptide P_646–755_ (located at VP1) and the peptide P_70–159_ (located at VP2), which did not induce cross-reactivity, were dissolved at 200 μg/mL in the same solution of the vaccine candidate and formulated as the positive or negative antigen to detect cross-reactivity [31] and injected intraperitoneally (i.p.) at 100 μL/mouse.

To evaluate the protective effect of maternal antibodies on neonatal mice, female mice aged 6 weeks were immunized. Each mouse was immunized with 10 μg fusion protein vaccine candidate, and their immunity was boosted 3 weeks later at the same dose and volume. One week later, female mice were allowed to mate. Sera for the determination of the neutralization titer to analyze cross-reactivity were collected from the female mice at 1 week, 4 weeks, 5 weeks, and 8 weeks after the first immunization. Six female mice were immunized for each group, including fusion protein vaccine candidate group, inactivated virus group and *E. coli* lysate group. For viral infection experiments, 12–15 neonatal mice born by immunized female mice were used for each group.

To assess the protective effect of active immunity of fusion protein vaccine candidate, 1-day-old neonatal mice were used for immunization i.p. at 50 μL/mouse. Immunity was boosted 1 week later with the same dose and volume. There were 12 neonatal mice were used for each group.

### Determination of neutralization titer in the sera of immunized mice

The CPE method was applied to determine the neutralization titer (NT) of mice sera from vaccinated female mice in infected RD cells. First, 100 μL of RD cell suspension with 2.0 × 10^4^ cells was added to each well in 96-well plates (Falcon) and incubated at 37 °C in a carbon dioxide incubator containing 5% CO_2_. Thereafter, series of two-fold dilutions of each mouse serum sample were prepared using DMEM with 2% FBS, and then 50 μL of each dilution was mixed with 50 μL of 200 TCID_50_ of FY0805 in DMEM supplemented with 2% FBS, followed by incubation at 37 °C for 1 h. Finally, the DMEM supernatant of overnight incubated RD cells was replaced by the total 100-μL mixture (4 wells/dilution), and CPE was observed after three consecutive days of culturing. NTs of sera were determined by calculating the highest dilution of serum that prevented infection in 50% of replicate inoculations [30].

### Immunohistochemical staining for analysis of cross-reactivity

Cross-reactivity of specific sera with human brain tissues was detected via immunohistochemical staining. Human cerebral and medullar of tissues were obtained from an adults and a fetus, both of whom died in accidents, and the usage of human brain tissues were permitted by Institutional Review Board of hospital [31].

In brief, the sera (1:500 dilution) from female mice immunized with 10 mM Glycine-NaOH buffer, inactivated FY0805, P_70–159_, P_646–755_, or the fusion protein vaccine candidate were used as primary antibodies. Frozen human brain sections were washed thrice with PBS after incubation with mice sera. Thereafter, the sections were incubated with HRP-conjugated goat anti-mouse IgG (1:5000 dilution, sigma) for 1 h at 37 °C. Finally, the sections were developed with 3–3′-diaminobenzidine and examined using a light microscope, as described previously [31].

### Evaluation of the effect of immunization via viral infection

Neonatal mice born from vaccinated female mice were exposed to a lethal EV71 challenge; every 1-day-old mouse was injected with 50 μL 10 LD_50_ (0.5 × 10^7^ TCID_50_/mL) or 200 LD_50_ (1 × 10^8^ TCID_50_/mL) of MP10 i.p., respectively. Nevertheless, the active immunized mice were subjected to a non-lethal dose EV71 challenge, and each two-week-old mouse was inoculated with 100 μL of MP10 (5 × 10^7^ TCID_50_/mL) i.p. The clinical scores were graded as described previously [32].

### Determination of viral load

To detect the viral RNA copies in tissues of infected-mice, quantitative polymerase chain reaction (qPCR) was performed [29]. In brief, total RNA was extracted from muscle tissue, using TRIzol reagent (Thermo). Reverse transcription was carried out immediately using random hexamers with a reverse-transcription kit (Promega). The cDNA was subjected to qPCR (QuantiTect SYBR Green RT-PCR kit, QIAGEN) for 40 cycles with a Roche LightCycler 3.5 system. The primers were EV71-S1 (5′-AGATAGGGTGGCAGATGTAATTGAAAG-3′) and EV71-A1 (5′-TAGCATTTGATGATGCTCCAATTTCAG-3′). A fragment corresponding to nucleotides 2462–2635 of FY0805 was used as standard with an adjusted concentration gradient (1 × 10^1^ copies/μL to 1 × 10^8^ copies/μL) to determine the viral RNA copies.

### Statistical analysis

Statistical analysis was performed using GraphPad Prism 6.0 (GraphPad Software, USA). Data of neutralizing titers (NTs) and viral RNA copies were presented as mean ± SD values. The significance of differences between two groups was assessed with the unpaired two-tailed Student t-test, while the data for more than two groups were assessed using Duncan’s multiple-range test followed by a one-way analysis of variance. Survival rates were analyzed via Kaplan–Meier analysis. Clinical scores were analyzed using Milcoxon test. A *p* value of < 0.05 was considered statistically significant (*p < 0.05, ** *p* < 0.001).

## Results

### Determination of the neutralization titers of fusion protein vaccine candidate

**Fig. 1:**
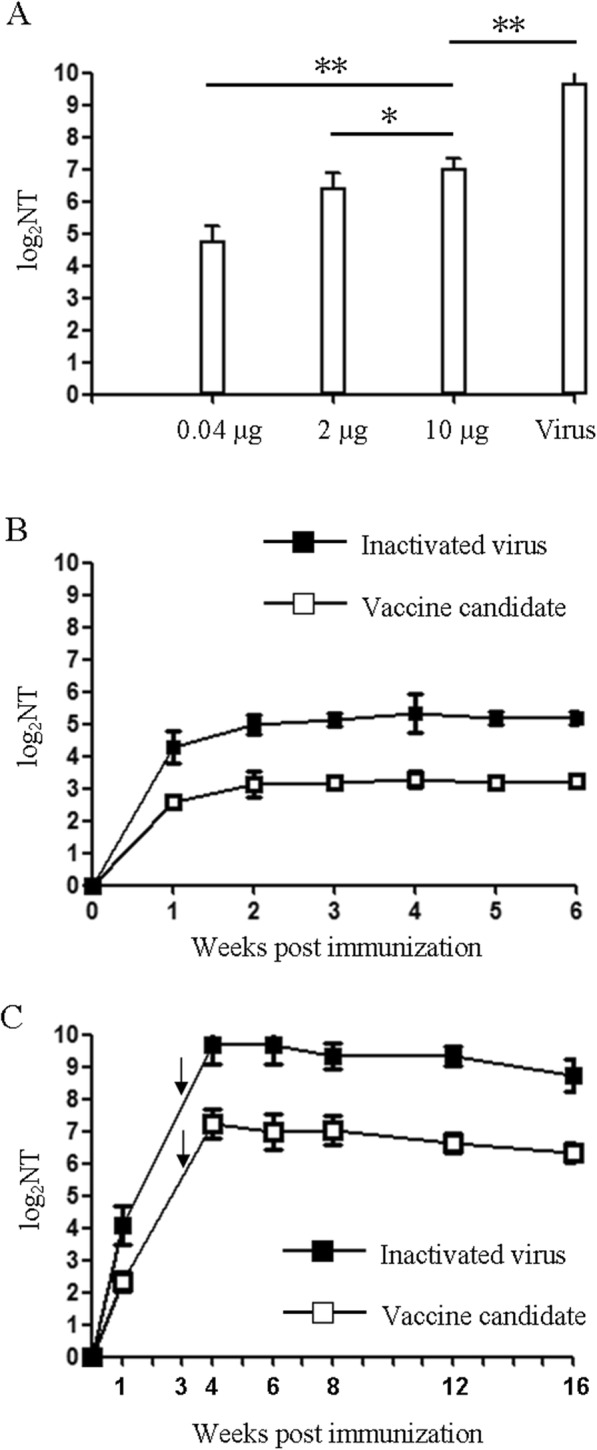
Determination of the neutralizing titers (NTs) of mice sera immunized with the vaccine candidate containing the fusion protein. **a** NTs of sera of mice immunized twice with 0.04 μg, 2 μg, and 10 μg vaccine candidate or inactivated virus were measured, respectively. *p < 0.05, ** *p* < 0.001. **b** The NTs of the sera of mice immunized once with 10 μg vaccine candidate or inactivated virus were measured temporally, respectively. **c** The NTs of sera of mice immunized twice with 10 μg vaccine candidate or inactivated virus were measured temporally, respectively. Arrows indicate the time of boosting. The data represent the mean ± SD (*n* = 6) values of two independent immunization experiments

We had previously generated a fusion protein with partial fragments of capsid proteins VP1, VP2, and VP3 of EV71, which were assembled into virus-like particles after purification and effectively elicit an immune response [18]. To further investigate the immune efficacy of the fusion protein as a vaccine candidate, we attempted three immunization dosages (10 μg, 2 μg, and 0.4 μg) to determine the optimal level of immunization. With an increase in the levels of the fusion protein, the neutralization activity of mouse serum obtained upon second immunization was significantly enhanced. Using the CPE method, the measured neutralization titers (NTs) were as follows: below 2^5^ for the 0.4 μg dosage group, above 2^6^ for the 2 μg dosage group, and approximately 2^7^ for the 10 μg dosage group (Fig. 1a). Based on the aforementioned results, we used 10 μg of fusion protein as a vaccine candidate for subsequent experiments. Meanwhile, booster immunization was necessary to gain higher NTs (Fig. 1b and Fig. 1c). The neutralization titer in the serum of mice immunized once peaked at 2^2^ after 2 weeks of immunization, and no decrease in immune response was observed after 6 weeks (Fig. 1b). For the mice with booster immunization, 4 weeks after the initial immunization, the NTs peaked at approximately 2^7^ a week after immunization and remained high (above 2^6^ 6 weeks post immunization) (Fig. 1c). The same situation occurred in the positive control, wherein mice immunized with the inactivated virus (Fig. 1), demonstrating that booster immunization induced a stronger humoral immune response.

### Cross-reactivity of the fusion protein vaccine candidate

**Fig. 2:**
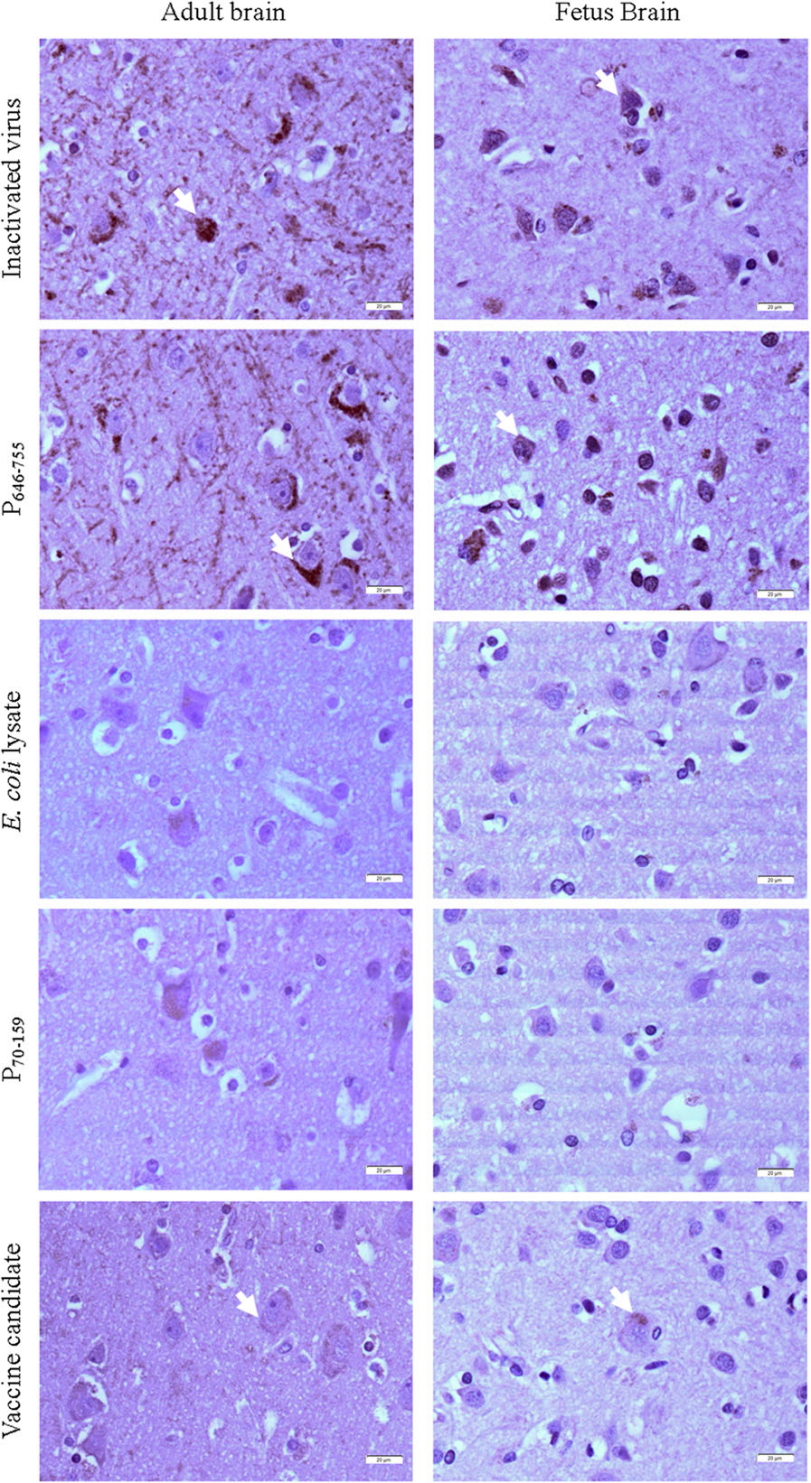
Immunohistochemical staining indicating weak cross-reactivity between mice sera immunized with the vaccine candidate containing the fusion protein with adult or fetus human brain tissues. Mouse sera immunized with inactivated virus or P_646–755_ were used as positive controls, and those immunized with *E. coli* lysate or P_70–159_ were used as negative controls. The cross-reactivity sites are denoted with white arrows. Magnification: 400×. The scale bars represented 20 μm in the figure

The serum of combined peptides vaccine comprising EV71 capsid protein peptides (P_70–159_, P_140–249_, P_324–443_, P_746–876_) did not exhibit strong cross-reactivity with human brain tissues [28]. However, it was necessary to check the reaction again when all peptides fused into one protein. The sera obtained from mice immunized with inactivated virus or P_646–755_ polypeptide (strong cross-reactivity peptide) were used as positive controls, while those immunized with *E. coli* lysate or P_70–159_ polypeptide (no cross-reactivity peptide) were used as negative controls [31]. Immunohistochemical staining revealed weak cross-reactivity when the sera of mice immunized with the fusion protein vaccine candidate were applied to adult or fetus human brain tissues (Fig. 2). This might be related to the weak cross-reactivity of peptides P_324–443_ and P_746–876_, which constituted the fusion protein [31].

### Protective effect of the maternal antibody on neonatal mice against EV71 infection

**Fig. 3:**
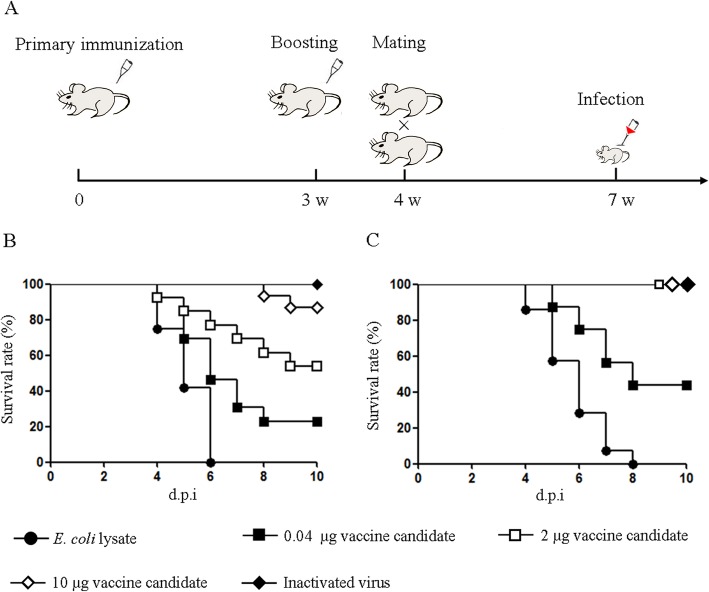
Protective effects of maternal antibodies from immunized mice on neonatal mice against EV71 infection. **a** A schematic representation of passive immunization and viral infection protocol. The protective effect of the fusion protein vaccine post 200 LD_50_ (**b**) or 10 LD_50_ (**c**) of viral infection were evaluated in 1-day-old neonatal mice (*n* = 12 ~ 15) born from immunized dams. Survival rates were analyzed via Kaplan–Meier analysis

The protective effect of the antibody transferred from immunized female mice to their newborn progenies against EV71 was experimentally assessed (Fig. 3a). Six-week-old female mice were vaccinated twice with fusion proteins (0.04 μg, 2 μg, or 10 μg, respectively), positive control (inactivated virus) and negative control (*E. coli* lysate), and their neonatal mice (1-day-old) were used for the subsequent experiment. No adverse effects of pre-mating immunity on normal pregnancy and production of female mice were found, and no significant differences in physiological status of their neonatal mice were found either, compared to normal female or neonatal mice (data not unshown).When infected with 200 LD_50_ of MP10 (a mouse-adapted EV71 strain), all negative control mice died 6 days-post-infection (d.p.i), while those in the positive control group still survived at 10 d.p.i. For the fusion protein immunization group, with an increase in the level of fusion protein, the survival rate of the neonatal mice also increased from 23% (0.04 μg) to 54% (2 μg) and to 87% (10 μg). The protective effect of maternal antibodies was more obvious when encountering a minor viral challenge with 10LD_50_. Except for the lowest dosage group (0.04 μg), immunization with the fusion protein completely inhibited EV71-mediated damage to neonatal mice. In contrast, mice in the negative control group died at 8 d.p.i, while those in the positive control group survived. Hence, similar to immunization with the inactivated virus vaccination, immunization of dams with the fusion protein effectively protected the neonatal mice against EV71 infection in cases of an infection at a low viral load.

### Protective effect of active immunization of mice against EV71 infection

**Fig. 4:**
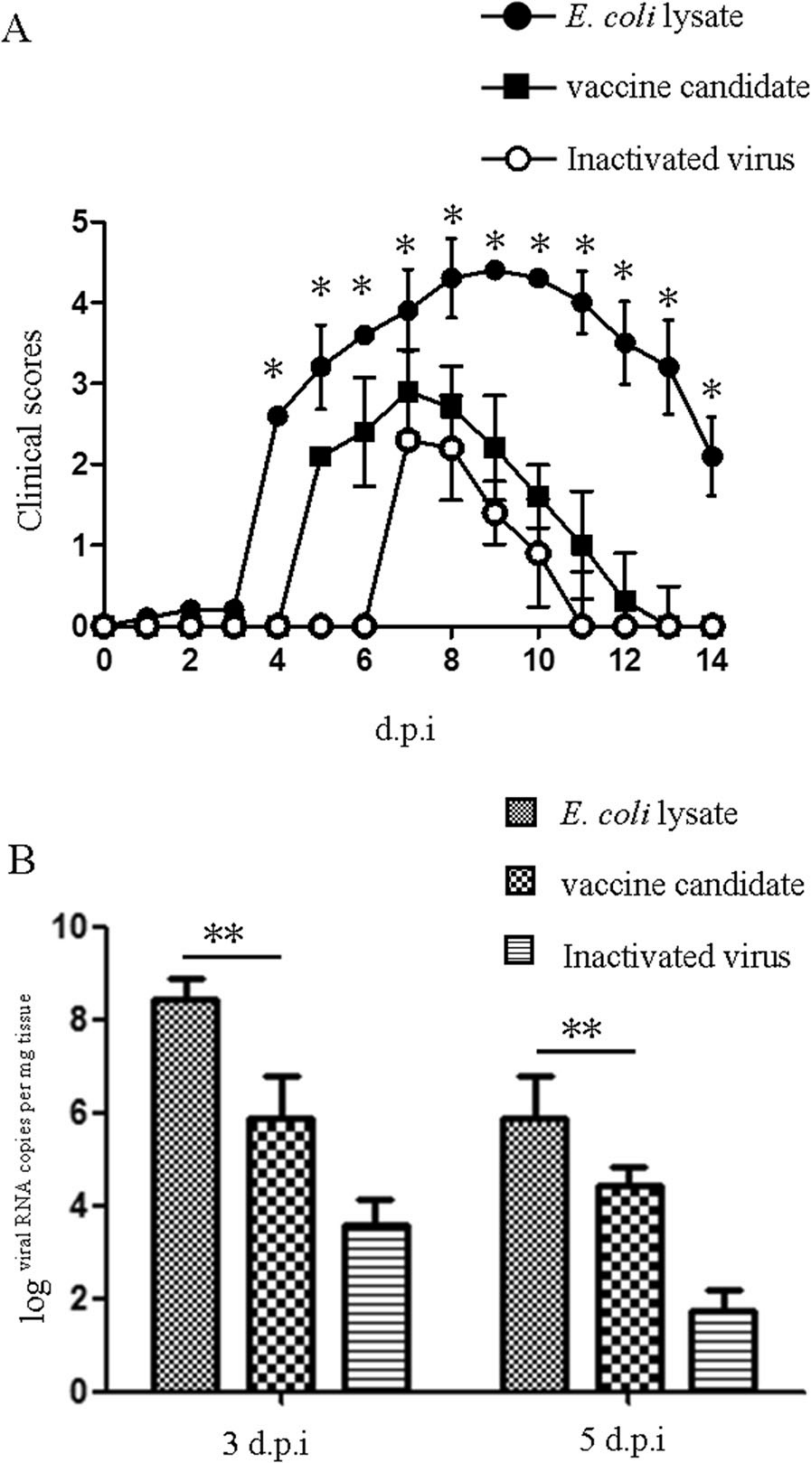
Protective effects of active immunization on mice against EV71 infection. **a** Clinical scores of mice immunized with 10 μg fusion protein-containing vaccine candidate upon sub-lethal EV71 infection (*n* = 12). Clinical scores were analyzed using Milcoxon test. **b** Viral RNA copies in skeletal muscle of EV71-infected mice were detected via quantitative reverse transcription polymerase chain reaction analysis (*n* = 6). The data represent the mean ± SD values of two independent immunization experiments. *p < 0.05, ** *p* < 0.001

We actively immunized normal 1-day-old mice twice to assess the protective effects of immunization with the fusion protein on new-born mice. One week after booster immunization, the vaccinated neonatal mice were subjected to a non-lethal dose of MP10 infection (5 × 10^7^ TCID_50_/mL). From the 4 d.p.i of symptom onset, the clinical scores of the mice in the fusion protein immunization group were significantly lower than those of the negative control group (Fig. 4a). Similar to the inactivated virus group, the average clinical score of mice in the fusion protein immunization group peaked at 4 d.p.i, and then gradually decreased until the symptoms alleviated on 13 d.p.i. Furthermore, viral replication was assessed in hind limb muscle tissue of mice, since the skeletal muscle was considered the primary site for viral amplification after EV71 infection (Fig. 4b). Viral RNA in the muscle of mice in the fusion protein group was approximately 10^6^ copies/mg on 3 d.p.i., which reduced to 10^4^ copies/mg at 5 d.p.i., being significantly lower than that of the negative control. In summary, the fusion protein exerted an excellent active immunoprotective effect on neonatal mice, albeit still inferior to the inactivated virus.

## Discussion

Prophylactic vaccines are extremely important to prevent the EV71 epidemic, especially for susceptible children. Owing to their complete immunoprotective activity, viral inactivated vaccines are the preferred treatment strategy. In mainland China, the development and clinical evaluation of inactivated EV71 vaccines and the promotion of vaccinations have progressed rapidly to prevent the prevalence of HFMD caused by EV71 infection [14–17, 33]. Meanwhile, to compensate for the low productivity deficits of inactivated vaccines, other strategies for vaccine development have been considered. Notwithstanding advancements in DNA vaccines [34], those containing recombinant protein subunits [35, 36], and those containing live attenuated viruses [37–39], VLPs have yielded more advantageous recombinant vaccines. VLP vaccines can fuse multiple antigenic sites together to completely expose epitopes on the surface, thereby inducing a more effective immune response [20, 22, 24–27, 40]. A novel EV71 VLP lacking VP4 (designated VLP_ΔVP4_) structurally mimics the 80S empty capsid, which is the end stage of EV71 uncoating, thereby exhibiting desirable immunogenicity and protection as a vaccine candidate [22]. A tetravalent VLP vaccine comprising CVA10-VLP, EV71-VLP, CVA16-VLP, and CVA6-VLP reportedly elicited antigen-specific lasting serum antibody responses with neutralization titers similar to those of their monovalent counterparts. Most importantly, passively transferred tetravalent vaccine-immunized sera showed efficient protective effects against single or mixed infections with EV71, CVA16, CVA10, and CVA6 viruses in mice [24]. HFMD is a multi-pathogen disease with more than 20 pathogens, and cases of CA16, CA10 and CA6 infection have increased year by year. Therefore, it’s necessary to develop multivalent vaccines, and VLP vaccines can be a potential choice.

In the previous study, we initially screened the EV71 viral capsid protein for peptide fragments inducing high neutralizing active antibodies, and selected the best combination; its genes were fused via recombinant DNA technology [18, 28]. Subsequently, using a highly efficient prokaryotic expression system, the fused protein was expressed as inclusion bodies and dissolved, purified, and refolded to form pentameric molecules, and finally assembled into spherical particles [18]. In the present study, we further assessed the immunoprotective effects of the fusion protein as a vaccine candidate. The highest dosage of the fusion protein (10 μg for immunization) and booster immunization induced specific serum antibodies with the highest neutralization titers, and the protective antibodies were transmitted from the dams to the progeny, thereby protecting the neonatal mice from EV71-mediated damage. Meantime, immunoprotective effects were also observed in neonatal mice after immunization with the fusion protein. Combined with the increased proliferation of splenocytes and secretion of cytokines (including IFN-γ, IL-2, IL-4, and IL-5), according to our previous report [18], the fusion protein effectively induced humoral and cell-mediated immune responses.

We previously reported that the sera of HFMD patients could react with human brain sections, suggesting potential cross-reactivity between antibodies of EV71 and brain tissues. Since EV71 induced IgG was permitted to enter into brain tissues by the increased permeability of the blood-brain barrier (BBB) after EV71 infection, cross-reactivity might result in neurological symptoms [31, 41, 42]. In addition, a common epitope (PPGAPKP) between capsid VP1 protein and human mediator complex subunit (MED25) was identified, suggesting the involvement of the immune response in neurological complications caused by EV71 infection [43]. The component fragment P_746–876_ and P_324–443_ of the fusion protein displayed weak positive cross-reactivity, hence probably resulting in weak cross-reactivity of the entire fusion protein. However, after immunizing neonates with the inactivated whole virus, no case of side reaction in brain was reported; hence, we presumed that the cross-reactivity may have resulted from the exposure of intracellular cross-antigens during the preparation of brain tissue sections, and it has been suggested that immune cross-reactivity should be monitored upon administration of the whole inactivated virus vaccine to children with neurological diseases.

The fusion protein vaccine used herein is derived from our optimization of a previous vaccine comprising a combination of peptides. Upon fusing the four gene fragments, the expression and purification of the four proteins was reduced to that of one corresponding protein, thereby saving time and production expenses. Moreover, compared with vaccines containing inactivated virus, the present vaccine candidate containing this fusion protein has a greater advantage with respect to its production and aggregation of dominant epitopes. Although its immunoprotective effect was inferior to that of the vaccine containing inactivated virus, it evidently exerted adequate immunoprotective effects via antibody production to prevent EV71-mediated damage to neonatal mice, thereby being a promising therapeutic alternative.

## Conclusions

Above all, the fusion protein combined by four gene fragments of EV71 capsid proteins was shown to play immunoprotective role in neonatal mice through maternal antibodies and active immunization, which remarkably alleviated EV71-mediated damage. Therefore, it’s suggested that the fusion protein is an applicable vaccine candidate in prevention of EV71 infection in addition to inactivated virus vaccine.

## Data Availability

All datasets used and analysed during the current study are available from the corresponding author on reasonable request.

## Abbreviations

HFMD: Hand, foot, and mouth disease
EV71: Enterovirus 71
VLPs: Virus-like particles
BBB: Blood-brain barrier
CNS: Central nervous system
NT: Neutralization titer
